# A comparative study on the efficacy of 10% hypertonic saline and equal volume of 20% mannitol in the treatment of experimentally induced cerebral edema in adult rats

**DOI:** 10.1186/1471-2202-11-153

**Published:** 2010-12-10

**Authors:** Hong-Ke Zeng, Qiao-Sheng Wang, Yi-Yu Deng, Wen-Qiang Jiang, Ming Fang, Chun-Bo Chen, Xin Jiang

**Affiliations:** 1 Department of Emergency & Critical Care Medicine, Guangdong General Hospital, Guangdong Academy of Medical Sciences, Guangzhou 510080, PR China; 2 Department of Critical Care Medicine, The First Affiliated Hospital, University of South China, Hengyang 421001, Hunan Province, PR China; 3 Graduate School, Southern Medical University, 1838 North Guangzhou Avenue, Guangzhou 510515, PR China

## Abstract

**Background:**

Hypertonic saline and mannitol are commonly used in the treatment of cerebral edema and elevated intracranial pressure (ICP) at present. In this connection, 10% hypertonic saline (HS) alleviates cerebral edema more effectively than the equal volume of 20% mannitol. However, the exact underlying mechanism for this remains obscure. This study aimed to explore the possible mechanism whereby 10% hypertonic saline can ameliorate cerebral edema more effectively than mannitol.

**Results:**

Adult male Sprague-Dawley (SD) rats were subjected to permanent right-sided middle cerebral artery occlusion (MCAO) and treated with a continuous intravenous infusion of 10% HS, 20% mannitol or D-[1-^3^H(N)]-mannitol. Brain water content (BWC) as analyzed by wet-to-dry ratios in the ischemic hemisphere of SD rats decreased more significantly after 10% HS treatment compared with 20% mannitol. Concentration of serum Na^+ ^and plasma crystal osmotic pressure of the 10% HS group at 2, 6, 12 and 18 h following permanent MCAO increased significantly when compared with 20% mannitol treated group. Moreover, there was negative correlation between the BWC of the ipsilateral ischemic hemisphere and concentration of serum Na^+^, plasma crystal osmotic pressure and difference value of concentration of serum Na^+ ^and concentration of brain Na^+ ^in ipsilateral ischemic hemisphere in the 10% HS group at the various time points after MCAO. A remarkable finding was the progressive accumulation of mannitol in the ischemic brain tissue.

**Conclusions:**

We conclude that 10% HS is more effective in alleviating cerebral edema than the equal volume of 20% mannitol. This is because 10% HS contributes to establish a higher osmotic gradient across BBB and, furthermore, the progressive accumulation of mannitol in the ischemic brain tissue counteracts its therapeutic efficacy on cerebral edema.

## Background

Cerebral edema is involved in many neurological diseases such as cerebral ischemia/hemorrhage, brain trauma and brain tumor or abscess [1]. Severe cerebral edema must be managed immediately to prevent brain herniation. Osmotic therapy is a cornerstone in nonsurgical management of intracranial pressure (ICP) induced by various cerebral edema. Its effectiveness depends on: 1) the integrity of the blood-brain barrier (BBB), 2) the reflection coefficient of the osmotic agent, and 3) the osmotic gradient created [2]. 20% mannitol, as a classical osmotic dehydrating agent, is widely used as a dehydrating agent in clinical patients with cerebral edema. However, because it may lead to hypovolemia, renal dysfunction [3,4], heart failure, electrolyte disturbance and other side effects, there are certain restrictions in clinical practice. In recent years, many studies have shown that 10% hypertonic saline (HS) may be effective in treatment of all kinds of cerebral edema and intracranial hypertension caused by various causes, including traumatic brain injury [5-8], cerebral stroke [9,10], subarachnoid hemorrhage [11], brain tumors [12], etc. Compared with mannitol, HS is more effective to ameliorate cerebral edema and intracranial hypertension [6-8,10,13-16]. Furthermore, HS remains significantly effective even in situations when use of mannitol had failed [10] or cerebral hernia [17]. However, the specific mechanism by which HS can ameliorate cerebral edema more effectively than equal volume of 20% mannitol remains unclear. This study sought to determine if 10% HS administration could decrease cerebral edema more effectively than 20% mannitol, and if so, whether the efficacy would be associated with a formation of Na^+ ^osmotic concentration gradient between blood and brain tissue. Additionally, we also investigated whether 20% mannitol could accumulate progressively in the ischemic cerebral tissue. We report here that 10% HS decreases brain water content (BWC) more effectively than equal volume of 20% mannitol. The results suggest that this may be attributed to a formation of Na^+ ^osmotic concentration gradient between blood and brain tissue by HS and progressive accumulation of 20% mannitol in the ischemic brain tissue.

## Methods

### Animals and experimental groups

Adult male Sprague-Dawley (SD) rats 250-300 g (body weight) (Experimental Animal Center of SUN YAN-SEN University, China) were randomly divided into a sham-operated group (*n *= 48), cerebral ischemic + normal saline treatment group (abbreviated: normal saline group) (*n *= 48), cerebral ischemic + 20% mannitol treatment group (abbreviated: 20% mannitol group) (*n *= 60), and cerebral ischemic + 10% HS treatment group (abbreviated: 10% HS group) (*n *= 48). Under aseptic surgical conditions, the tail vein was cannulated to facilitate the intravenous (i.v.) infusion of 20% mannitol, 10% HS or normal saline. Rats in normal saline group, 20% mannitol group and 10% HS group were subjected to permanent right-sided middle cerebral artery occlusion (MCAO). In the sham-operated group, the rats were anesthetized with 5% Ketamine (FuJian GuTian Pharmaceutical Co., Ltd., China) following which the right common carotid artery (CCA) was exposed but not subjected to MCAO. At 6 h following MCAO, the rats in sham-operated group and normal saline group were treated with normal saline (0.3 ml/h). At the same time point, the rats in 20% mannitol group and 10% HS group were treated with a continuous intravenous infusion (0.3 ml/h) of 20% mannitol or 10% HS, respectively, via the tail vein until the end of the experiment. The rats in sham-operated group, normal saline group, 20% mannitol group and 10% HS treatment group were further subdivided into four subgroups according to different treatment times: 2, 6, 12 and 18 h. In addition, 12 of the rats in 20% mannitol group were also subdivided correspondingly into four subgroups: 2, 6, 12 and 18 h following the onset of treatment with 20% D-[1-^3^H(N)]-mannitol (NET101005MC., PerkinElmer, Inc. USA). In this connection, the rats were treated with a continuous intravenous infusion (50 μCi/h) of D-[1-^3^H(N)]-mannitol via the tail vein until the end of the experiment. The number of rats killed at various time points in different groups is shown in Table 1. Animal handling and experiments were approved by Institutional Animal Care and Use Committee, Guangdong Province, China.

**Table 1:** Number of rats killed at various time points in different groups

Groups	BWC	Concentration of brain Na^+^	Blood parameters	Autoradiography and concentration of D-[1-^3^H(N)]-mannitol
Sham-operated group				
2 h	6	6	8	0
6 h	6	6	8	0
12 h	6	6	8	0
18 h	6	6	8	0
Normal saline group				
2 h	6	6	8	0
6 h	6	6	8	0
12 h	6	6	8	0
18 h	6	6	8	0
20% mannitol group				
2 h	6	6	8	3
6 h	6	6	8	3
12 h	6	6	8	3
18 h	6	6	8	3
10% HS group				
2 h	6	6	8	0
6 h	6	6	8	0
12 h	6	6	8	0
18 h	6	6	8	0

### Focal brain ischemia animal model

In rats subjected to MCAO, they were fasted overnight prior to surgery, but were allowed free access to water. The rats were anesthetized with 5% Ketamine (40 mg/kg). Rectal temperature was maintained between 37 and 37.5°C with a heating lamp throughout the surgical procedures. Focal brain ischemia was induced by the intraluminal suture MCAO method as described previously [18,19,13]. Briefly, the right CCA, internal carotid artery (ICA), and external carotid artery (ECA) were exposed through a midline incision of the neck. A 4-0 headend spherical nylon suture was used as an occluder and was inserted via the CCA. For the CCA occlusion route, the proximal portions of the right CCA and ECA were ligated with 5-0 surgical sutures, and the occluder was inserted through an arteriotomy of the right CCA 3 mm below the carotid bifurcation. The occluder was advanced into the ICA 17 to 19 mm beyond the carotid bifurcation. Mild resistance indicated that the occluder was properly lodged in the middle cerebral artery (MCA) and thus blocked the blood flow to the artery. Sham-operated rats were subjected to the surgical procedures except for MCAO. Neurologic examinations were performed 6 h after the onset of occlusion. The neurologic findings were scored on a five-point scale [18]: 0, no neurologic deficit; 1, a mild focal neurologic deficit (failure to extend left forepaw fully); 2, a moderate focal neurologic deficit (circling to the left); and 3, a severe focal deficit (falling to the left); 4, no spontaneous motor activity (the rats did not walk spontaneously and had a depressed level of consciousness). The rats with neurologic deficit score of 1 to 3 were considered as an effective model.

### Assessment of ischemic hemispheric brain edema

Rats were killed at the end of the experiment by decapitation under deep anesthesia. The brain was quickly removed and gently blotted to remove small quantities of adsorbent moisture and were dissected through the interhemispheric fissure into ipsilateral ischemic and contralateral hemispheres. Brain edema was estimated by comparing wet to dry weight ratios [20,21]. Tissues were weighed with a scale to within 0.001 mg. Dry weight of the entire ischemic hemispheres was determined after heating the tissue for 3 days at 100°C in a drying oven. Tissue water content was then calculated as % H_2_O = (1-dry wt/wet wt) × 100% [20,21].

### Assessment of concentration of brain Na^+ ^of ischemic hemisphere, serum Na^+ ^and plasma crystal osmotic pressure

Brain tissue was removed as described above. Normal saline at 2 ml/g ischemic hemisphere brain tissue was then added. The tissue was homogenated by power-driven in the ice, followed by centrifugation at 12,000 rev/min for 5 min. Finally, concentration of brain Na^+ ^of ischemic hemisphere was detected by automatic biochemical analyzer (UniCel DxC 800 Synchron, Beckman Coulter, Inc., USA) in 500 μL supernatant. In addition, Four mL blood samples were drawn from rats under deep anesthesia. The blood samples were then centrifuged (3,000 rev/min for 5 min) after standing 2 h. The concentration of serum Na^+ ^and plasma crystal osmotic pressure were detected by an automatic biochemical analyzer (UniCel DxC 800 Synchron, Beckman Coulter, Inc., USA) with 500 μL supernatant.

### Assessment of the non-ischemic and ischemic hemisphere D-[1-^3^H(N)]-mannitol content by autoradiography and liquid scintillation method

#### Method for autoradiography

Rats were killed at the end of the experiment by decapitation under deep anesthesia. They were perfused transcardially with saline and the brain removed immediately. Coronal frozen sections of the brain of 30 μm thickness at the level of the optic chiasma were cut and fixed for 30 sec with anhydrous methanol. Brain sections were immersed in nuclear emulsion N-4 (Fujifilm Corporation, Japan) for 30 sec and then exposed for four weeks in the darkroom. Subsequently the sections were developed, fixed and fully dried. The number of silver grains of the peri-infarct brain tissue and the corresponding non-ischemic brain tissue was calculated by counting six randomly selected microscopic fields in sections obtained from each rat (*n *= 3) at × 20 objective by a blinded observer.

#### Liquid scintillation method

Fifty mg of the peri-infarction brain tissue as well as the corresponding non-ischemic brain tissue at the level of the optic chiasma level was quickly removed. Two hundred μL perchloric acid and 200 μL 30% hydrogen peroxide were added successively in the brain tissue. The specimen was reacted in the 80°C incubator for 30 min resulting in a homogeneous liquid. After the liquid was cooled to room temperature, 200 μL of the liquid was transferred to special bottles for liquid scintillation. In the latter, 2 mL scintillation fluid and 2 mL ethanol were added, respectively. The reaction mixture was shaken for a few sec turning it into a clear liquid. The concentration of the D-[1-^3^H(N)]-mannitol was then detected by liquid scintillation analyzer (Tri-Carb 2900TR, PerkinElmer, Inc. USA).

#### Data analysis and statistics

All data were analyzed using the SPSS13.0 statistical software. Different statistical methods were applied according to types of data. Values were expressed as mean ± standard deviation (± SD). Univariate-factor measurement data was analyzed by one-way ANOVA, but two-factor measurement data was analyzed by two-way classification ANOVA. Multiple comparisons were analyzed by LSD method if the data was homogeneity of variance, otherwise they were analyzed by Dunnett's T3 method. Bivariate correlation analysis of measurement data was analyzed by Pearson correlation analysis. The difference was considered statistical significance when *P *< 0.05.

## Results

### Ipsilateral ischemic hemispheric BWC

**Figure 1:**
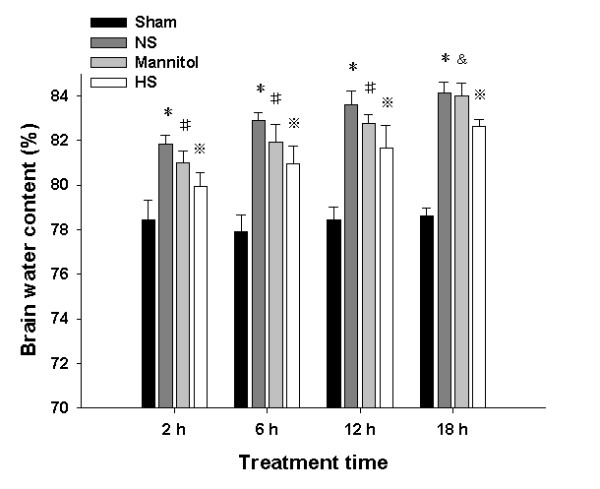
**Ipsilateral ischemic hemispheric BWC**. BWC(%) in the ipsilateral ischemic hemisphere of the normal saline group, 20% mannitol group and 10% HS group at 2, 6, 12 and 18 h following permanent MCAO and the corresponding sham operated group. Bar graph shows that the percentage of BWC is significantly increased in the ipsilateral ischemic hemisphere of the normal saline group, 20% mannitol group and 10% HS group at various time points when compared with the sham operated group (*^#&^*P *< 0.05). The percentage of BWC decreases significantly in the ipsilateral ischemic hemisphere at 2, 6 and 12 h following treatment with 20% mannitol or 10% HS compared with the normal saline group (^#^*P *< 0.05, ^※^*P *< 0.05). At 18 h, compared with the normal saline group, the percentage of BWC in the 20% mannitol does not decrease significantly *(^&^P *> 0.05). However, the BWC of 10% HS group continues to decrease significantly (^※^*P *< 0.05). BWC in the ipsilateral ischemic hemispheres of the 10% HS group at 2, 6, 12 and18 h decreases significantly when compared with the 20% mannitol group(^※^*P *< 0.001).

Analysis of ischemic hemispheric brain edema revealed that BWC in the ipsilateral ischemic hemispheres of the normal saline group, the 20% mannitol group and the 10% HS group at 2, 6, 12 and18 h following permanent MCAO increased significantly when compared with that in the sham operated group (*^#※&^*P *< 0.05) (Figure 1). Compared with the normal saline group, BWC in the ipsilateral ischemic hemispheres of the 20% mannitol group and the 10% HS group at 2, 6 and12 h decreased significantly (^#※^*P *< 0.05). At 18 h, however, the BWC of 20% mannitol group was not significantly different in comparison with the normal saline group (^&^*P *= 0.551). On the other hand, the BWC of 10% HS group continued to decrease and was significantly different from the normal saline group (^※^*P *< 0.001). BWC in the ipsilateral ischemic hemispheres of the 10% HS group at 2, 6, 12 and18 h decreased significantly when compared with the 20% mannitol group (^※^*P *< 0.001) (Figure 1).

### Concentration of brain Na^+ ^of ischemic hemisphere, serum Na^+ ^and plasma crystal osmotic pressure

**Figure 2:**
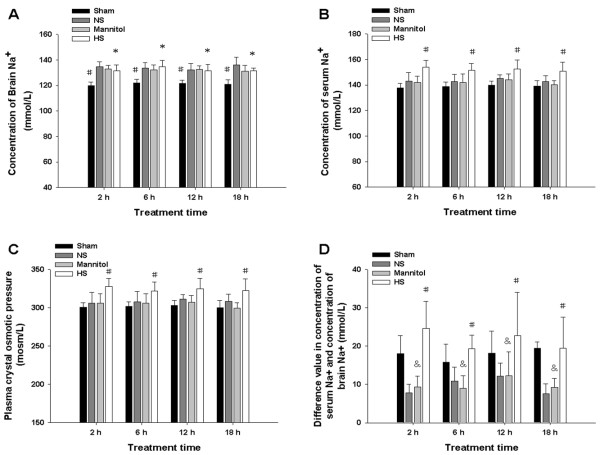
**Plasma crystal osmotic pressure, brain Na^+ ^concentration, serum Na^+ ^concentration and difference value of both**. Na^+ ^concentration of the ipsilateral ischemic hemispheres (A), serum Na^+ ^concentration (B), plasma crystal osmotic pressure (C) and difference value in concentration of serum Na^+ ^and concentration of brain Na^+ ^in ipsilateral ischemic hemisphere (D) of the normal saline group, the 20% mannitol group and 10% HS group at 2, 6, 12 and18 h following permanent MCAO as well as matching sham operated group

Analysis of concentration of brain Na^+ ^of ischemic hemisphere revealed that in the normal saline group, 20% mannitol group and the 10% HS group at 2, 6, 12 and 18 h following permanent MCAO, it was increased significantly when compared with that in the sham operated group (^#^*P *< 0.001) (Figure 2A). The concentration of brain Na^+ ^in the 10% HS was not increased significantly when compared with that of the normal saline group and 20% mannitol group (*P > 0.05) (Figure 2A). Analysis of concentration of serum Na^+ ^revealed that in the 10% HS group at 2, 6, 12 and18 h following permanent MCAO, it was increased significantly when compared with that in the sham operated group, the normal saline group and the 20% mannitol group (^#^*P *< 0.001) (Figure 2B). The concentration of serum Na^+ ^in the 10% HS was not significantly different between the various time points (*F *= 0.381, *P *= 0.767) (Figure 2B). Analysis of plasma crystal osmotic pressure revealed that in the 10% HS group at 2, 6, 12 and18 h following permanent MCAO, it was increased significantly when compared with that in the sham operated group, the normal saline group and the 20% mannitol group (^#^*P *< 0.01) (Figure 2C). The plasma crystal osmotic pressure in the 10% HS did not differ significantly between various time points (*F *= 0.341, *P *= 0.796) (Figure 2C). Furthermore, additional analysis of the difference value in concentration of serum Na^+ ^and Na^+ ^in ipsilateral ischemic hemisphere revealed that in the 10% HS group at 2, 6, 12 and 18 h following permanent MCAO, it was increased significantly when compared with the normal saline group and the 20% mannitol group(^#^*P *< 0.05) (Figure 2D). Compared with the sham operated group, the difference value of concentration of serum Na^+ ^and brain Na^+ ^in ipsilateral ischemic hemisphere of the 10% HS group at 2, 6, 12 and 18 h following permanent MCAO was not significantly different (^#^*P *> 0.05). Moreover, there was no difference between the normal saline group and the 20% mannitol group at 2, 6, 12 and 18 h following permanent MCAO (^&^*P *> 0.05) (Figure 2D). At the same time, Correlation analysis revealed that there was negative correlation between the BWC of the ipsilateral ischemic hemisphere and concentration of serum Na^+ ^(r = -0.811, -0.865, -0.863, -0.911, respectively; All *P *< 0.05) and plasma crystal osmotic pressure (r = -0.813, -0.888, -0.887, -0.915, respectively; All *P *< 0.05) in the 10% HS group at 2, 6, 12 and 18 h following permanent MCAO.

### Autoradiography

**Figure 3:**
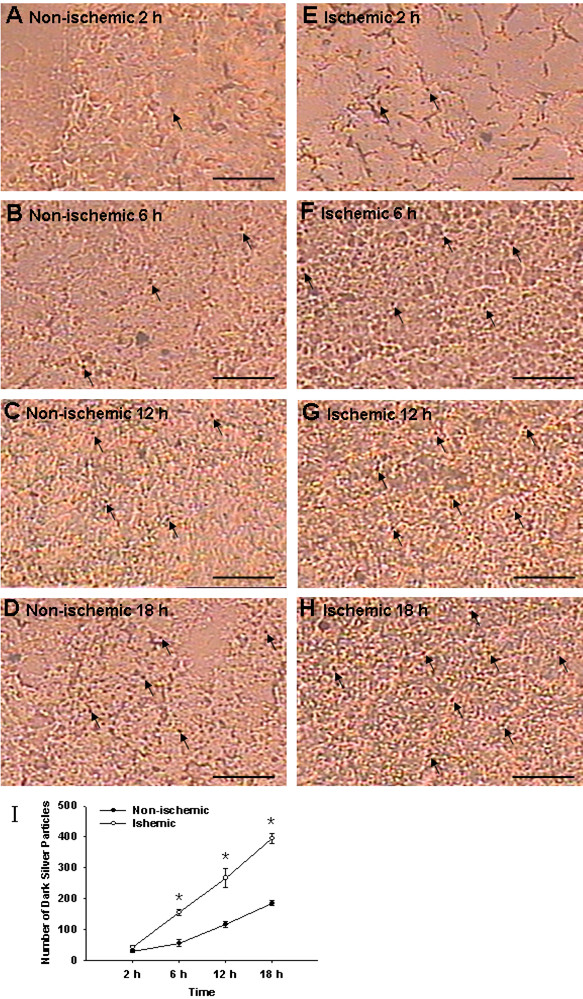
**Autoradiography in the peri-infarction brain tissue and the corresponding areas in the non-ischemic tissue**. Autoradiography shows the number of black silver particles in the peri-infarction brain tissue. The number of black silver particles increases from 2 to 18 h after administration with D-[1-^3^H(N)]-mannitol (E, F, G, H) in comparison with the corresponding areas in the non-ischemic tissue (A, B, C, D) being more marked at 12 and 18 h (G, H). The number of dark silver particles also increases in the non-ischemic brain tissue from 2 to 18 h (A, B, C, D), notably at 12 and 18 h (C, D), but the number of particles is evidently less than that in the ischemic brain tissue at corresponding time points. Figure 3I shows a significant increase in the black silver particles in the peri-infarction brain tissue at 6, 12 and 18 h following permanent MCAO. Scale bars: A-H, 20 μm.

Analysis of autoradiography revealed that the number and distribution of dark silver particles in autoradiography may reflect indirectly the content and distribution of D-[1-^3^H(N)]-mannitol. Dark silver particles were sparsely distributed in the peri-infarction brain tissue at 2 h following administration with D-[1-^3^H(N)]-mannitol (Figure 3E). The silver grains appeared to increase in number from 6 to 18 h (Figure 3F, G, H). Compared with 2 h, the dark silver particles were more numerous at 12 and 18 h (Figure 3G, H) suggesting that mannitol was progressively accumulated in the ipsilateral infarcted region. Likewise, the incidence of the silver particles increased continuously in the corresponding non-ischemic brain tissue from 2 to 18 h (Figure 3A, B, C, D), notably at 12 and 18 h (Figure 3C, D). Results of actual particle count showed that the number of dark silver particles in the pre-infarction brain tissue and the corresponding areas in the non-ischaemic tissue increased from 2 to 18 h after administration with D-[1-^3^H(N)]-mannitol (*P *< 0.01). The number of the silver particles in the non-ischemic brain tissue was less than that in the ischemic brain tissue at 6, 12 and 18 h (*P < 0.01) (Figure 3I).

### Concentration of D-[1-^3^H(N)]-mannitol in the peri-infarction brain tissue and corresponding non-ischemic brain tissue

**Figure 4:**
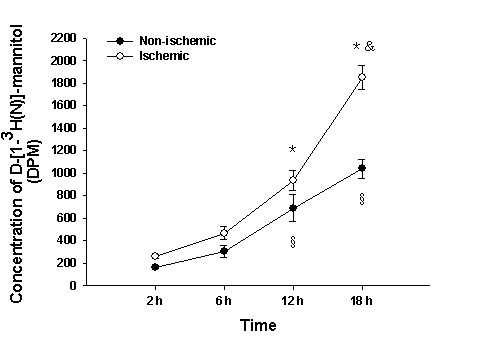
**Concentrations of D-[1-^3^H(N)]-mannitol in the ischemic and non-ischemic brain tissue**. Concentrations of D-[1-^3^H(N)]-mannitol in the ischemic and non-ischemic brain tissue at 6, 12 and 18 h following permanent MCAO. Note the progressive increase in concentration of D-[1-^3^H(N)]-mannitol in the peri-infarction brain tissue continuously from 2 to 18 h after administration with D-[1-^3^H(N)]-mannitol as detected by liquid scintillation analyzer.

Analysis of liquid scintillation method revealed that the concentration of D-[1-^3^H(N)]-mannitol in the peri-infarction brain tissue increased progressively from 2 to 18 h after administration with D-[1-^3^H(N)]-mannitol. It increased notably at 18 h in comparison with that at 2, 6 and 12 h (^&^*P *< 0.001) (Figure 4). This indicates that mannitol continued to accumulate in the ipsilateral infarcted region. The concentration of D-[1-^3^H(N)]-mannitol was also progressively increased in the corresponding non-ischemic hemisphere brain tissue from 2 to 18 h. Compared with 2 h, the concentration of D-[1-^3^H(N)]-mannitol did not increase significantly at 6 h in the peri-infarction brain tissue and corresponding non-ischemic brain tissue (*P *= 0.064) (Figure 4). However, increase at 12 and 18 h in comparison with 6 h was significant (^§^*P *< 0.001) (Figure 4). The concentration of D-[1-^3^H(N)]-mannitol in the peri-infarction brain tissue increased significantly at 12 and 18 h when compared with corresponding non-ischemic hemisphere brain tissue (*P < 0.05) (Figure 4).

## Discussion

HS alleviates cerebral edema and lowers ICP by establishing an osmotic gradient between the intracellular and intravascular space [22]. It has been widely used for the treatment of cerebral edema and elevated intracranial pressure (ICP) resulting from cerebral infarction [13,23], hemorrhage [24] or traumatic brain injury [25,26]. Studies have shown that when compared with equal volume of mannitol, HS solutions may be more effective in lowering elevated ICP [27]. HS at 7.5% showed a higher increase in serum osmolality at 15 min after administration than 20% mannitol [28]. But, cerebrospinal fluid, mean arterial pressure and central venous pressure were not different between the 7.5% HS and 20% mannitol treatment [28]. In both pediatric and adult traumatic brain injury (TBI), HS has been used effectively to reduce elevated ICP which is refractory to mannitol administration [25,26]. This study has shown that BWC in the ipsilateral ischemic hemispheres of the 20% mannitol group and the 10% HS group at 2, 6 and 12 h following permanent MCAO decreased significantly when compared with that in the normal saline group. However, when compared with BWC of the normal saline group at 18 h, the BWC of 20% mannitol group was not reduced, but the BWC of 10% HS group was decreased significantly. BWC in the ipsilateral ischemic hemispheres of the 10% HS group at 2, 6, 12 and18 h decreased significantly when compared with the 20% mannitol group. BWC can reflect the degree of brain edema [29,30]. Hence, the present results suggest that 10% HS is more effective in treatment of cerebral edema when compared with the 20% mannitol.

Na^+ ^ions increased commonly in the cerebral edema region, with a concomitant decrease in K^+ ^ions [31,32]. The increase in Na^+ ^ions may be attributed to reduced brain tissue spaces resulted from cerebral edema and, furthermore, the Na^+ ^ions are not reabsorbed because of obstruction of CSF reflux [32]. In addition, the activation of corresponding ion channels such as NKCC1 plays an important role in increasing the concentration of Na^+ ^ions in ischemic cerebral tissue [33-35]. Another possible explanation would be that Na^+ ^ions enter the cerebral parenchyma via increased BBB permeability caused by BBB breakdown. This study has shown that the concentration of Na^+ ^ions in ipsilateral ischemic hemisphere in the normal saline group, 10% HS group and 20% mannitol group increases significantly. However, Na^+ ^concentration in the 10% HS group was not significantly different from the normal saline group and the 20% mannitol group. These results demonstrated that 10% HS administration did not cause an abnormal accumulation of Na^+ ^in the brain tissue. The reason may be that 10% HS does not aggravate BBB breakdown [36].

The formula for plasma crystal osmotic pressure is as follows: plasma crystal osmolality (mosm/L) = 2 [Na ^+^(mmol/L) + K ^+^(mmol/L)] + BUN (mmol/L) + Glu (mmol/L). It is suggested from the formula that Na ^+ ^ions play an important role in the maintenance of plasma crystal osmotic pressure. In this connection, if the concentration of serum Na^+ ^increases, the plasma crystal osmotic pressure will be inevitably increased. In addition, the Na^+ ^ions have been reported to play a major role in the maintenance of homeostasis and osmotic pressure balance inside and outside cells [37,38]. The same is true in the brain in which Na^+ ^ions imbalances are bound to lead to the osmotic pressure imbalance inside and outside cells and the cerebrovascular system [39]. Therefore, the difference value of concentration of serum Na^+ ^and concentration of brain Na^+ ^reflect indirectly the osmotic pressure gradient inside and outside the BBB. The present results have shown that the concentration of serum Na^+ ^or plasma crystal osmotic pressure in the 10% HS group at 2, 6, 12 and 18 h increased significantly when compared with that in the other groups. Moreover, compared with other groups, the difference value of concentration of serum Na^+ ^and concentration of brain Na^+ ^in ipsilateral ischemic hemisphere in 10% HS group was increased significantly. Further analysis showed that not only there was negative correlation between the BWC of the ipsilateral ischemic hemisphere and concentration of serum Na^+ ^or plasma crystal osmotic pressure, but also there was negative correlation between the BWC of the ischemic hemisphere and the difference value of concentration of serum Na^+ ^and concentration of brain Na^+ ^in ipsilateral ischemic hemisphere in the 10% HS group at 2, 6, 12 and 18 h following permanent MCAO. Therefore, it is speculated that one of the reasons that 10% HS can ameliorate the cerebral edema more effectively than 20% mannitol may be because it can maintain a higher osmotic pressure gradient inside and outside the BBB.

This study has also shown that D-[1-^3 ^H (N)]-mannitol tends to accumulate in the ischemic cerebral hemisphere and the non-ischemic cerebral hemisphere, being most pronounced at 18 h. It is noteworthy that the effect of 20% mannitol in ameliorating the cerebral edema was weakened significantly at 18 h. A possible explanation for this would be that mannitol leaks through the disrupted BBB continuously and accumulates in the brain parenchyma, so that the osmotic pressure gradient inside and outside of the BBB is decreased or even reversed. This would weaken its effect in ameliorating the cerebral edema and may even aggravate the latter. Kaufmann and Cardoso [40] and Cho et al [41] reported that mannitol accumulated constantly in the injured cerebral hemisphere when it was used repeatedly by the intravenous route, so that the osmotic pressure gradient inside and outside the BBB is reversed, thus exacerbating the cerebral edema. The reason that mannitol accumulates in the non-ischemic brain tissue may be related to increase of BBB permeability resulting from a variety of inflammatory mediators induced by ischemia, hypoxia and oxidative stress [42,43] and opening of BBB by itself [44]. In an experimental animal study, it was reported that not only BBB breakdown, but also BWC of ipsilateral hemisphere increased significantly after healthy adult female Wistar rats were injected with mannitol via the left jugular vein [44]. A possible mechanism related to this may be shrinkage of vascular endothelial cells and opening of endothelial tight junctions by mannitol [45,46]. Undoubtedly, abnormal accumulation of 20% mannitol in the pre-ischemic cerebral hemisphere and the non-ischemic cerebral hemisphere offset an osmotic pressure gradient inside and outside the BBB induced by mannitol. These also help to explain the facts that mannitol is less effective to ameliorate cerebral edema and intracranial hypertension when compared with HS.

## Conclusions

This study has shown that 10% HS is more effective in alleviating cerebral edema compared with equal volume of 20% mannitol. The following are possible mechanisms: 1) a higher osmotic pressure gradient is established by 10% HS across the BBB; 2) mannitol tends to accumulate progressively in the brain tissue and more severely in the ischemic cerebral hemisphere than the non-ischemic cerebral hemisphere with continuous intravenous injection of 20% mannitol. Undoubtedly, this will counteract the therapeutic efficacy of 20% mannitol on cerebral edema.

## Abbreviations

BBB: blood-brain barrier; BWC: brain water content; CCA: common carotid artery; CSF: cerebrospinal fluid; ECA: external carotid artery; HS: hypertonic saline; ICP: intracranial pressure; ICA: internal carotid artery; MCA: middle cerebral artery; MCAO: middle cerebralartery occlusion; SD: Sprague-Dawley.

## Competing interests

The authors declare that they have no competing interests.

## Authors' contributions

ZHK carried out the design of the study and performed the statistical analysis. WQS carried out assessment of the non-ischemic and ischemic hemisphere D-[1-^3^H(N)]-mannitol content by autoradiography and liquid scintillation method, collected data and drafted the manuscript. DYY participated in the design of the study and drafted the manuscript. JWQ carried out assessment of ischemic hemispheric brain edema. FM participated in assessment of concentration of brain Na^+ ^of ischemic hemisphere, serum Na^+ ^and plasma crystal osmotic pressure. CCB performed the statistical analysis. JX participated in making the focal brain ischemia animal model. All authors read and approved the final manuscript.
